# Altered Glycosylation of Human Alpha-1-Acid Glycoprotein as a Biomarker for Malignant Melanoma

**DOI:** 10.3390/molecules26196003

**Published:** 2021-10-03

**Authors:** Dávid Virág, Tibor Kremmer, Kende Lőrincz, Norbert Kiss, Antal Jobbágy, Szabolcs Bozsányi, Lili Gulyás, Norbert Wikonkál, Gitta Schlosser, Adina Borbély, Zsófia Huba, Borbála Dalmadi Kiss, István Antal, Krisztina Ludányi

**Affiliations:** 1 Department of Pharmaceutics, Semmelweis University, Hőgyes Endre utca 7., H-1092 Budapest, Hungary; virag.david@pharma.semmelweis-univ.hu (D.V.); famkremmer@gmail.com (T.K.); gyorizsofireka@gmail.com (Z.H.); kiss.borbala@pharma.semmelweis-univ.hu (B.D.K.); antal.istvan@pharma.semmelweis-univ.hu (I.A.); 2 Department of Dermatology, Venereology and Dermatooncology, Semmelweis University, Mária utca. 41., H-1085 Budapest, Hungary; lorincz.kende@med.semmelweis-univ.hu (K.L.); kiss.norbert@med.semmelweis-univ.hu (N.K.); jobbagyantal@gmail.com (A.J.); bozsanyi.szabolcs@med.semmelweis-univ.hu (S.B.); gulyaslili1998@gmail.com (L.G.); wikonkal.norbert@med.semmelweis-univ.hu (N.W.); 3 MTA-ELTE Lendület Ion Mobility Mass Spectrometry Research Group, Institute of Chemistry, Faculty of Science, ELTE Eötvös Loránd University, Pázmány Péter sétány 1/A, H-1117 Budapest, Hungary; gitta.schlosser@ttk.elte.hu (G.S.); adina.borbely@ttk.elte.hu (A.B.)

**Keywords:** alpha-1-acid glycoprotein, biomarker, glycosylation, hydrophilic interaction chromatography, linear discriminant analysis, mass spectrometry, melanoma

## Abstract

A high-resolution HILIC-MS/MS method was developed to analyze anthranilic acid derivatives of *N*-glycans released from human serum alpha-1-acid glycoprotein (AGP). The method was applied to samples obtained from 18 patients suffering from high-risk malignant melanoma as well as 19 healthy individuals. It enabled the identification of 102 glycan isomers separating isomers that differ only in sialic acid linkage (α-2,3, α-2,6) or in fucose positions (core, antenna). Comparative assessment of the samples revealed that upregulation of certain fucosylated glycans and downregulation of their nonfucosylated counterparts occurred in cancer patients. An increased ratio of isomers with more α-2,6-linked sialic acids was also observed. Linear discriminant analysis (LDA) combining 10 variables with the highest discriminatory power was employed to categorize the samples based on their glycosylation pattern. The performance of the method was tested by cross-validation, resulting in an overall classification success rate of 96.7%. The approach presented here is significantly superior to serological marker S100B protein in terms of sensitivity and negative predictive power in the population studied. Therefore, it may effectively support the diagnosis of malignant melanoma as a biomarker.

## 1. Introduction

Malignant melanoma (MM) is a skin tumor that arises from melanocytes responsible for melanin production and its transfer to keratinocytes. Formerly, MM was considered a rare tumor to affect mainly the elderly population, but during the past five decades, its worldwide incidence dramatically increased with a rate greater than that of most malignancies. Although it represents less than 5% of all cutaneous malignancies, its high mortality rate and severe metastatic potential establish the need to develop specific and sensitive methods to recognize the disease and provide less invasive alternatives to traditional diagnostic procedures including histopathological and immunohistochemical techniques [1,2]. To date, the available serological biomarkers, such as lactate dehydrogenase and S100B protein, have been of prognostic value only [3]. They show a better correlation with advanced clinical stages as well as the presence of metastases and have limited diagnostic usefulness [3].

Glycosylation is a post-translational modification affecting stability, structural integrity, and functional properties in more than half of eukaryotic proteins [4,5]. Recent studies have also demonstrated that in various physiological and/or pathological states (pregnancy, inflammation, autoimmune diseases, and cancer), the sugar composition and structure of circulating glycoproteins are changed, and the appearance of irregular molecule variants can be detected [6,7,8,9]. Among several natural glycoconjugates, alpha-1-acid glycoprotein (AGP), also known as orosomucoid, is one of the characteristic, highly glycosylated (~45% carbohydrate content) and frequently studied protein fraction of human serum, with a molecular mass of 41–43 kDa [10]. AGP has five *N*-glycosylation sites on the polypeptide chain (asparagine location 15, 38, 54, 75, 85), having complex (antennary) glycan side chains and providing a great structural variability to the molecule [10]. AGP is produced primarily by the liver; however, various extrahepatic tissues, including cancer cells, have also been reported to express AGP under certain physiological and pathological conditions [11]. Altered glycosylation, such as changes in branching, fucosylation, and sialylation of the molecule was described in several cancer types, highlighting the relevance of AGP as a promising biomarker for malignant diseases [12,13,14,15].

The success of developing new glycan structure-based biomarkers relies greatly on the selection of proper chemo/biometrical methods for interpreting the large amount of information embedded in the sugar composition of glycoproteins [16,17]. Linear discriminant analysis (LDA) is a widely used technique for dimensionality reduction and pattern recognition applications. Within a three-step process, including calculation of between-class variation, within-class variation, and construction of a lower-dimensional space, LDA maximizes class separability and is able to predict which group an element belongs to [18]. In combination with bioanalytical methods, LDA can, therefore, serve as a potent tool for diagnostic and even progression monitoring purposes.

Given that the diagnostics of MM is deficient in proper bio/tumor markers, the present work aimed to conduct a comprehensive clinical study focusing on the alterations recognized in the glycosylation pattern of AGP in melanoma. In the current study, oligosaccharide side chains of human serum AGP isolated from melanoma patients as well as from healthy individuals were released by enzymatic digestion, labeled with anthranilic acid (AA), and investigated comparatively using a hydrophilic interaction chromatography–tandem mass spectrometric (HILIC-MS/MS) method. Results of the bioanalytical measurements evaluated by LDA provide foremost information on irregular glycosylation of AGP in melanoma patients with potential use as a disease marker.

## 2. Results and Discussion

### 2.1. HILIC-MS/MS

**Figure 1:**
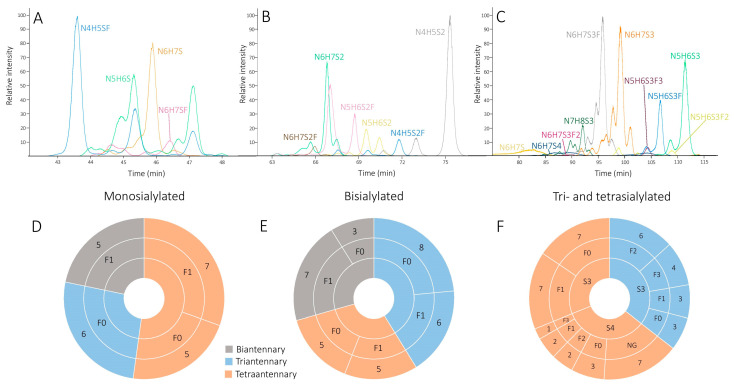
Extracted ion chromatograms and structural features of the major monosialylated (**A**,**D**), bisialylated (**B**,**E**), tri- and tetrasialylated (**C**,**F**) AGP glycan isomers. N refers to N-acetylglucosamine, H to hexoses (galactose and mannose), G to galactose, S to sialic acid, and F to fucose units. The digits after the letters refer to the number of each residue that makes up the molecule. The number of isomers corresponding to the same glycan composition is indicated in the outer layer of the diagram.

Serum AGP of 18 high-risk melanoma patients and 19 healthy individuals were isolated. The carbohydrate content of each individual sample was released enzymatically and derivatized with AA, which provides high-resolution chromatographic separation and enhances ionization efficiency of glycans in the negative ion mode [19,20]. Derivatives were subsequently analyzed by a newly developed and optimized HILIC-MS/MS method to reveal alterations in the glycosylation pattern that may be used to differentiate between healthy and pathological samples. The extracted ion chromatograms in Figure 1 show that glycans were eluted in three well-separated peak clusters according to the number of sialic acid residues as follows: 43.49–47.15 min, monosialylated; 61.36–75.31 min, bisialylated; 83.46–112.60 min, tri-, and tetrasialylated oligosaccharides. These findings indicate that the separation of glycans in HILIC is primarily affected by the number of sialic acid residues, while the number of antennas, fucose units, and other structural features such as extra *N*-acetyllactosamine units on the chain have only minor effects on retention. Processing of data acquired in LC-MS experiments resulted in the identification of 102 complex type *N*-glycan isomers, which, according to our present knowledge, is the largest number ever detected in AGP. It was advanced by the high-resolution chromatographic method, as well as the relatively large amount of glycoprotein processed that allowed the separation and detection of glycan isomers even with very low abundance. AGP is considered a sialoglycoprotein, where all the glycans detected contain at least one sialic acid residue, and several mono-, bi-, and trifucosylated oligosaccharides, as well as glycans containing *N*-acetyllactosamine (NG) chain elongation, were also identified (see Figure 1).

Evaluation of LC-MS/MS experiments enabled a reliable characterization of 39 of the most abundant glycan isomers. Ion fragments were annotated based on the nomenclature proposed by Domon and Costello [21]. Under optimized conditions, tandem MS spectra were dominated by Y-, Z- and B-type ion fragments facilitating sequence characterization; however, cross-ring fragmentation resulting in A-ions were also observable. The large number of isomers identified (in some cases up to 8) is primarily due to sialic acid residues connected with α-2,3 or α-2,6 linkage to the antennas, resulting in great structural variability of isomeric glycans. Diagnostic fragments appearing on the spectra clearly demonstrated the presence of each linkage type. We used singly charged ^0,4^A_2_–CO_2_ (*m*/*z* 306.12) fragment ion to detect α-2,6 and linkage and B_2_–CO_2_ (*m*/*z* 408.15) fragment for α-2,3 sialic acids, as suggested by several authors [22,23,24]. Baseline separation of bisialylated biantennary glycan (N4H5S2) eluting in three chromatographic peaks between 70.53 and 75.31 min was achieved. Diagnostic fragments confirmed that isomer 1 was fully α-2,3 sialylated, 2 contained α-2,3, as well as α-2,6 linkages and isomer 3 had only α-2,6-linked sialic acids. These findings suggest that sialic acid linkage types are closely related to the elution order since the increase in the number of α-2,6 linkages resulted in higher retention in HILIC mode. It is important to note that although some overlap has occurred, it seems that the chromatographic separation was able to provide satisfactory resolution of isomers even in more branched glycans.

**Figure 2:**
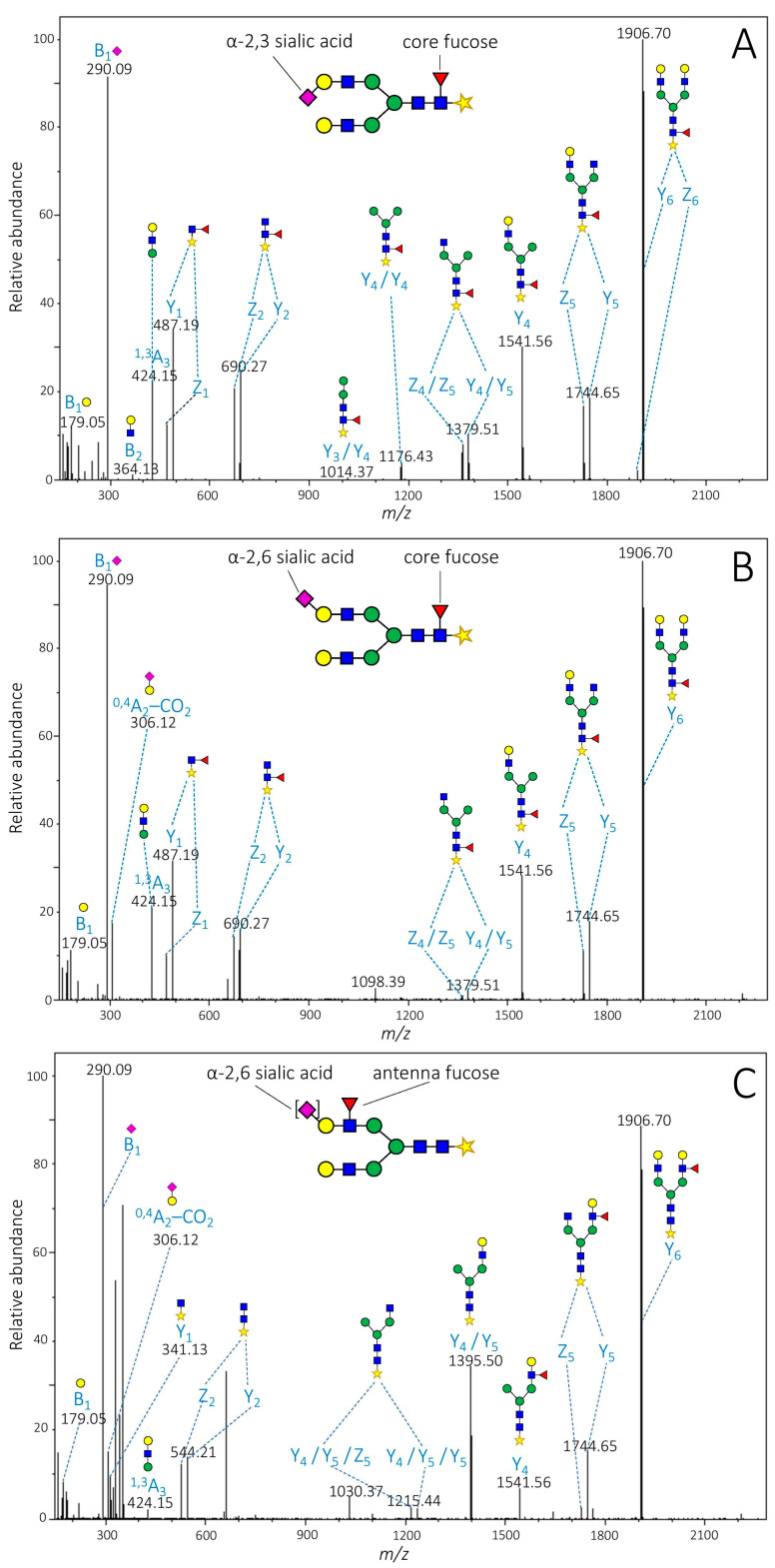
MS/MS spectra of isomers 1 (**A**), 3 (**B**), and 5 (**C**) of AA derivatized biantennary glycan N4H5SF obtained from a pooled healthy control sample.

Biantennary glycan N4H5SF bearing only one sialic acid, while eluting in five baseline-separated chromatographic peaks between 43.49 and 47.11 min indicates the presence of fucose isomerism. Assignation of the MS/MS spectra showed that isomer 1 had α-2,3 linked sialic acid, while isomer 3 and 5 were α-2,6 sialylated (Figure 2). MS/MS spectrum of isomer 1 contained a set of Y (and corresponding Z) ion fragments diagnostic for core fucosylation including Y_1_ (*m*/*z* 487.19), Y_2_ (*m*/*z* 690.27), Y_3_/Y_4_ (*m*/*z* 1014.37) and Y_4_/Y_4_ (*m*/*z* 1176.43) (Figure 2A). Isomer 3 exhibited *m*/*z* 487.19 and *m*/*z* 690.27 fragments only, probably due to low intensity (Figure 2B). On the other hand, Y_1_ (*m*/*z* 341.13) Y_2_ (*m*/*z* 544.21) fragments of isomer 5 had not any fucose units, demonstrating the absence of core fucosylation. In addition, ion fragments Y_4_/Y_5_/Y_5_ (*m*/*z* 1233.45) and Y_4_/Y_5_ (*m*/*z* 1395.51) of isomer 5 are indicators of antenna fucosylation, considering that antenna fucose is bound weaker to the sugar backbone than core fucose, therefore preferably removed during fragmentation (Figure 2C) [24,25,26]. Another biantennary glycan, N4H5S2F, eluting between 67.93 and 73.50 min, was found to have two isomers with core fucosylation. Considering that isomers containing core fucose were present both in healthy and cancerous samples, it can be ruled out that they are products of the malignant transformation. Consequently, the current study does not support previous findings that AGP is exclusively antenna fucosylated [27,28]. Table S1 summarizes the retention times, mass accuracies, as well as fucose positions and sialic acid linkage types (where available) of the isomers, that were identified.

**Figure 3:**
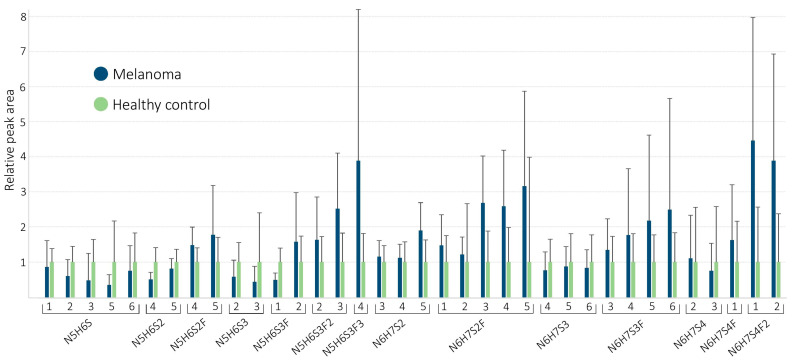
Averaged relative peak areas of the isomers demonstrating characteristic changes between the groups. Values of the melanoma group were normalized to the control group. Relative standard deviations (RSD) are also indicated.

To compare samples from affected and control individuals, extracted ion chromatograms of the glycans were obtained, and peak areas of all isomers were determined. In order to minimize differences arising from sample preparation in terms of intensity, the relative peak area of each isomer was expressed as the percentage of the sum of the peak areas in a given sample. To further reduce the complexity of data and enhance visual comparison, relative peak areas of glycan were averaged within the two classes (malignant and control), then resulting values of the cancerous sample group were normalized to the control group. As Figure 3 illustrates, overexpression of fucosylated glycans was recognized in the melanoma group, especially in more branched chains. An increase in some triantennary (N5H6S3F, N5H6S3F2, N5H6S3F3) and tetraantennary oligosaccharides (N6H7S4F, N6H7S4F2) also revealed that the extent of overexpression is seemingly proportional to the number of fucose units connected to the sugar backbone. This indicates directly the crucial relevance of fucosylation in the malignant process [29,30]. Downregulation in the nonfucosylated counterparts (N5H6S2, N5H6S3, N6H7S3) of some glycans observed may be a consequence of increased fucosylation in melanoma samples.

While Figure 3 represents clear tendencies, monitoring changes in the ratio of single isomers led to a classification of limited effectiveness only due to the large inter-individual variabilities (see RSD values on the graphic bar). This means that assigning a cut-off value to the isomers and categorizing the samples accordingly resulted in 75% (or worse) classification success. Although it represents some improvement over the performance of the S100B protein (see the next section), the application of more advanced chemometric methods handling multiple variables and enhancing pattern recognition seems to be required.

### 2.2. Statistical Analysis of Clinical Samples

**Figure 4:**
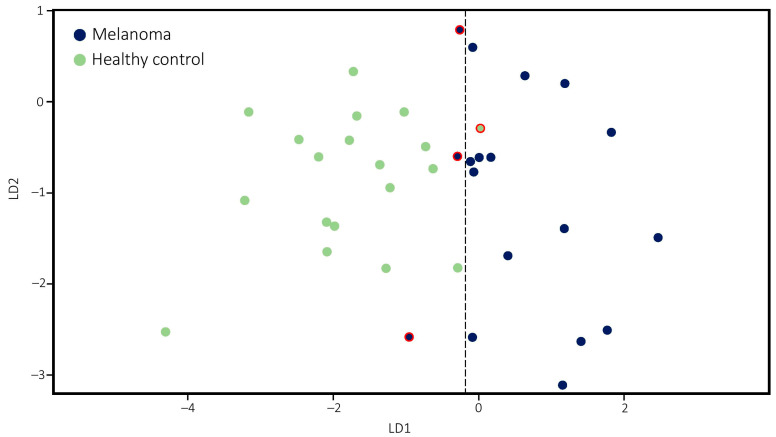
Classification of the 37 samples using LDA. Healthy individuals (green), melanoma samples (blue), and misclassifications (red outline). For better visualization, an additional category having zero effect on classification was added to the model.

Results obtained from LC-MS measurements were evaluated by linear discriminant analysis (LDA), applying a linear combination of continuous independent variables to predict two or more categories as dependent variables. Relative peak areas of individual isomers, glycans (calculated as the sum of the relative peak area of all isomers corresponding to the same glycan), and isomer peak area ratios of the same glycan were taken into account when searching for independent variables to develop the method. A general principle of LDA is that the number of independent variables should be less than one-third of the total number of samples studied (in the present case less than 12), otherwise the small sample size problem may occur [12,18]. Additionally, in order to maximize the class separability of the method, variables with the highest discriminatory power have to be selected. This was performed by determining Fisher’s index (F), calculated as the ratio of between and within-group variances. The higher the F value is, the better class separability is provided. Table 1 details that 10 of the most meaningful F values were associated with four glycans, reinforcing the above-mentioned trends that upregulation of fucosylated (N6H7S2F, F = 0.85) and downregulation of certain nonfucosylated glycans (N5H6S2, F = 0.99) may play a key role in the classification of malignant and control samples. Moreover, it occurred that isomers of the same glycan do not equally contribute to the differentiation, as isomer 4 (F = 1.20) and isomer 5 (F = 0.46) of N5H6S2, as well as isomer 3 (F = 0.98) of N6H7S2F, provided higher F value than others. When isomers of the same glycan were compared directly, an increased ratio of the later-eluting compound in the disease samples was observed in many cases. This was most pronounced in the ratio of isomers 4 and 5 (F = 0.74) of N5H6S2, 1, 2 (F = 0.27) of N5H6S3F, 3, 5 (F = 1.12) and 4, 5 (F = 0.97) of N6H7S2, as well as isomers 2 and 3 (F = 1.23) of N6H7S2F. In complete agreement with the elution order of glycans in HILIC, MS/MS experiments confirmed that in the case of N5H6S3F and N6H7S2, this is related to an increased degree of α-2,6 linked sialic acid, as reported in cancerous samples previously (Figures S1 and S2) [15,31]. The 10 observations with the highest discriminatory power were considered as independent variables, while patient classes (melanoma, control) were dependent variables. In the most crucial part of the evaluation, LDA transforms the features into a lower-dimensional space, which maximizes the ratio of the between-class variance to the within-class variance providing maximum separability of the classes [18]. Graphical representation of the lower-dimensional space can be seen in Figure 4, showing that malignant and healthy samples are well separated, and only a small overlap can be recognized between the classes, resulting in the misidentification of three melanoma samples as healthy and one control sample as melanoma, which means that the classification power of the model is 89.2%.

The effect of variables on the categorization was also assessed. This was accomplished by re-creating the method 10 times but omitting out a variable constantly. Without exception, this led to diminished performance manifesting typically in the misidentification of four melanoma samples at least as healthy. Therefore, all the variables played a crucial role in classification power. Evaluating the performance of the LDA method is of critical relevance. This was carried out following the “leave-1/3-out” approach of cross-validation, where a trainset of 25 (~2/3 of the samples) randomly selected samples were generated. The remaining 12 samples (~1/3 of the samples) were considered as unknowns. Categories of the test samples were then predicted by the LDA method developed according to the trainset. The whole process was repeated 10 times that allowed us to analyze 120 randomly selected samples. Taken together, we were able to accurately identify 52 out of 55 melanomas and 64 out of 65 control samples, to reach cross-validation with an outstanding 96.7% success rate. Results of the classification model and the cross-validation were compared with the S100B protein in Table 2 from the most important aspects of biomarker assessment. At a cut-off value of 0.15 µg/L, S100B provided the correct classification of 18 out of 19 controls, but 12 out of 18 MM samples were mistakenly identified as healthy individuals. Although S100B protein is highly specific and has a high positive predictive value, AGP was shown to be markedly superior to this biomarker in the population studied as regards the sensitivity and negative predictive power. Therefore, the results presented here indicate that the changes recognized in the sugar composition of AGP at the glycan structural level can serve as a powerful biomarker in the diagnosis of MM. 

## 3. Materials and Methods

### 3.1. Materials

Vacuette® 9 mL tubes without additives were obtained from Greiner Bio-One International GmbH (Kremsmünster, Kirchdorf an der Krems District, Austria). LC-MS grade methanol, acetonitrile (ACN) and formic acid (FA), HPLC grade absolute ethanol, chloroform and ammonia (32%), analytical grade ortho-boric acid, and sodium acetate trihydrate were purchased from VWR Chemicals (Radnor, PA, USA). 1,3-bis[tris(hydroxymethyl)methylamino]propane (bis-tris propane) (98+%) was purchased from Alfa Aesar (Haverhill, MA, USA). Alpha-1-acid glycoprotein from human plasma (≥99%), molecular biology grade K_2_HPO_4_, sodium dodecyl sulfate (SDS), ß-mercaptoethanol (ß-ME), and ethylenediaminetetraacetic acid (EDTA) were obtained from Sigma Aldrich (St. Louis, MO, USA). Sodium cyanoborohydride (95%) was purchased from Acros Organics (Geel, Belgium). Fractogel TMEA-650 (M) was obtained from Merck (Darmstadt, Germany). AA (≥99%) and molecular biology grade NaCl were obtained from Fisher Scientific (Hampton, NH, USA). Peptide:*N*-glycosidase F (PNGase F) was purchased from Roche Diagnostics (Mannheim, Germany). Sephadex G-25 Superfine gel was purchased from GE Healthcare (Chicago, IL, USA). Water of Milli-Q (MQ) purity prepared by Simplicity® Water Purification System (Merck Millipore, Burlington, MA, USA) was used.

### 3.2. Patients and Sample Handling

Peripheral blood samples without anticoagulants were collected from 60 melanoma patients treated at the Dermatology Department of Semmelweis University, Budapest. In total, 19 healthy volunteers served as controls. After spontaneous clotting at room temperature, serum samples were isolated by centrifugation and kept at −20 °C until use. Clinical diagnosis of melanoma was performed by dermoscopy that was followed by surgical removal and histopathological evaluation of the primary parameters of the tumor. A thorough clinical workup with proper imaging methods was carried out to establish the TNM classification [32]. In addition, serological marker, serum S100B protein level, was determined in all cases. Patients (18) in the advanced stage of the disease and those with high-risk malignant melanoma were selected as representative cases for the study [33]. Table S2 details the clinical characteristics of high-risk melanoma patients.

In sample preparation, non-miscible solvent extraction of 5 mL of serum samples was carried out according to our method described previously [34]. Briefly, 1 vol of MQ water and 15 vol of a chloroform–methanol mixture (2:1) were added to the serum. After 45 min of vigorous shaking in a water bath (0–4 °C), 2.5 vol of MQ water was added to the mixture and shaken further for 15 min. The supernatant (upper phase) was collected, and protein content was precipitated with twice the volume of ice-cold absolute ethanol and centrifuged (4000 rpm, 20 min, 4 °C). Separation of AGP from interfering components (mainly albumin) was performed by ion-exchange chromatography using a Fractogel TMAE EMD-650 M column (5 mm i.d. × 60 mm) where mobile phase A was 25 mM bis-tris propane (pH = 7.5) and B was 25 mM bis-tris propane, with 350 mM NaCl (pH = 9.5). The gradient program at a flow rate of 1.5 mL/min was as follows: 0–2.5 min, 0% B; 2.51–20.0, 50% B; 20.1–27.0, 60% B; 27.1–32.0, 100% B; 32.1–37.0, 100% B; 37.1–38.0, 0% B; 38.1–45.0, 0% B. AGP fraction collected between 11.3 and 16.8 min was desalted by gel filtration using a Sephadex G-25 column (2500 mm i.d. × 15,000 mm) eluted with MQ water at a flow rate of 2 mL/min. AGP was collected between 12.0 and 22.0 min. Samples were then freeze-dried and stored at 2–8 °C. Oligosaccharide side chains were released from the peptide chain based on the method of Elliott et al. [35]. A total of 2 mg of AGP was dissolved in 600 µL of buffer containing 50 mM K_2_HPO_4_ and 2 mM EDTA (pH 7.2). Then, 12 µL of 5% SDS and 16 µL of 10% ß-ME were added to the solution, and the glycoprotein was denaturated for 10 min at 100 °C. After cooling the sample for 10 min at −20 °C, SDS precipitates were eliminated by centrifugation (14,800 rpm, 3 min, 4 °C). Afterward, 6 µL (6 IU) of PNGase F was added to the supernatant and incubated for 24 h at 37 °C. Peptides were eliminated by adding 6 mL of absolute ethanol to the sample and centrifuging (4000 rpm, 5 min, 4 °C). The supernatant was then dried under a nitrogen stream (30 min, 40 °C). For derivatization of released glycans, 60 mg of AA and 40 mg of cyanoborohydride were dissolved in 2 mL of reagent solution containing 4% of sodium acetate trihydrate and 2% orthoboric acid in methanol. The oligosaccharides were dissolved in the reagent and incubated at 80 °C for 60 min according to Anumula et al. [36]. The samples were then diluted 1 to 3 with MQ water and purified by gel chromatography, lyophilized, and kept at 2–8 °C until analysis.

### 3.3. HILIC-MS/MS and Data Analysis

Chromatographic separations were performed on a Dionex UltiMate 3000 UHPLC system (Thermo Scientific, Bremen, Germany). The analytes were separated on a Phenomenex Luna^®^ NH2 column (3.0 mm i.d. × 250 mm, 5 µm, 100 Å). The autosampler and the column were maintained at 4 °C and 50 °C, respectively. Mobile phase A was 200 mM ammonium formate (pH = 3.50) and B was ACN. The gradient program at a flow rate of 0.4 mL/min was as follows: 0–3 min, 70% B; 3–95 min, 70–5% B; 95–110 min, 5% B; 110–111 min, 5–70% B; 111–130 min, 70% B. The freeze-dried samples were dissolved in 200 µL of mobile phase A and 100 µL was injected. Eluted glycan derivatives were monitored with a Q Exactive Focus Orbitrap MS (Thermo Scientific, Bremen, Germany) equipped with an electrospray ion source operating in negative mode. Optimized MS parameters were as follows: spray voltage, 2.8 kV; source temperature, 320 °C; sheath gas flow, 32 psi; aux gas flow, 7 psi; sweep gas flow, 0 psi; automatic gain control (AGC) target, 3e6; max injection time, 200 ms. MS spectra were collected between the mass range of 1000–2500 *m/z* at a resolution of 17500 FWHM. For data-dependent MS/MS experiments, five inclusion lists were established based on the exact mass measurements. Fragmentation was achieved using higher-energy C-trap dissociation (HCD), with a normalized collision energy of 35%. MS/MS spectra were acquired between 150 and 3000 *m/z* at a resolution of 17,500 FWHM. MS/MS measurements of pooled melanoma and control samples were carried out in separate runs. Data were collected and analyzed by Xcalibur 3.1. software (Thermo Scientific, Bremen, Germany). Xcalibur QualBrowser, Microsoft Excel (Microsoft Corporation, Redmond, WA, USA), and RStudio (RStudio, Boston, MA, USA) were used for data processing and statistical analysis.

## 4. Conclusions

In this study, a HILIC-MS/MS method was developed and optimized for characterizing AA labeled *N*-glycans released from human serum AGP. The high-resolution HILIC method, in combination with the optimized MS conditions, allowed the identification of the highest number of glycan isomers (102) ever detected in human serum AGP. This application also provided valuable information of critical relevance to discriminate sialic acid linkage (α-2,3 or α-2,6) and fucose positional (core, antenna) isomers. For the first time, our method was successfully used when samples obtained from 18 melanoma patients and 19 control individuals were compared. We demonstrated that changes in the glycan composition of human serum AGP are relevant in the diagnosis of malignant melanoma. Differences between the healthy and cancerous groups were manifested mainly in the upregulation of fucosylated glycan isomers, as well as in the downregulation of their nonfucosylated counterparts. In the case of some glycan structures, shifts in the ratios of certain isomers demonstrably related to an increased degree of α-2,6 sialic acid linkage were also observed. Considering that input variables of the statistics with the highest classification power were associated with overexpression of single isomers as well as isomer ratios of the same glycan, the application of high-resolution separation techniques is of crucial importance for revealing the information embedded in the sugar composition. However, none of these characteristics alone were meaningful enough to provide satisfactory differentiation between the melanoma and healthy samples due to the large inter-individual variabilities. On the other hand, statistical evaluation of the analytical data using LDA resulted in advanced classification power. When it comes to the comparative assessment of serological biomarkers, our approach outperformed significantly the S100B protein with respect to sensitivity and negative predictive power. In conclusion, the results presented here verify that the changes appearing in the glycosylation pattern of human serum AGP may serve as powerful support in the diagnosis of malignant melanoma as a biomarker.

## Figures and Tables

**Table 1:** The glycans and their characteristics with the corresponding F values that were found to be most meaningful in the classification.

Glycan	Discriminating Factor	Observation in MM	Fisher’s Index
N5H6S2	Isomer 4/Isomer 5	↓	0.74
	Isomer 4	↓	1.20
	Isomer 5	↓	0.46
	All Isomers	↓	0.99
N5H6S3F	Isomer 1/Isomer 2	↓	0.27
N6H7S2	Isomer 3/Isomer 5	↓	1.12
	Isomer 4/Isomer 5	↓	0.97
N6H7S2F	Isomer 2/Isomer 3	↓	1.23
	Isomer 3	↑	0.98
	All isomers	↑	0.85

**Table 2:** Comparison of AGP and S100B protein in terms of sensitivity, specificity, positive and negative predictive power for MM. Results of the cross-validation are also indicated.

	Sensitivity (%)	Specificity (%)	Positive Predictive Value (%)	Negative Predictive Value (%)
S100B Protein	33.3	94.7	85.7	60.0
AGPC lassification	83.3	94.7	94.7	85.7
AGPC ross-Validation	94.6	98.5	98.1	95.5

## Data Availability

The data used to support the findings of this study are available from the corresponding author upon request.

## Supplementary Materials

The following are available online. Table S1: The glycan isomers identified in the melanoma and control samples, Table S2: Clinical characteristics of the melanoma patients, Figure S1: MS/MS spectra of isomers 1 (A) and 2 (B) of triantennary glycan N5H6S3F, Figure S2: MS/MS spectra of isomers 3 (A), 4 (B), and 5 (C) of tetraantennary glycan N6H7S2.

## Sample Availability

Samples of the compounds are not available from the authors.
