# Supplementary material for: Altered Glycosylation of Human Alpha-1-Acid Glycoprotein as a Biomarker for Malignant Melanoma
Source: Molecules. 2021 Oct 3;26(19):6003. doi: 10.3390/molecules26196003 (PMC8513036; doi:10.3390/molecules26196003)
Supplement: Supplementary file 1 [file molecules-26-06003-s001.zip › molecules-1402142-supplementary.pdf]

Article

Altered Glycosylation of Human Alpha-1-acid Glycoprotein as a Biomarker for Malignant Melanoma

Dávid Virág[1], Tibor Kremmer[1], Kende Lőrincz[2], Norbert Kiss[2], Antal Jobbágy[2], Szabolcs Bozsányi[2], Lili Gulyás[2], Norbert Wikonkál[2], Gitta Schlosser[3], Adina Borbély[3], Zsófia Huba[1], Borbála Dalmadi Kiss[1], István Antal[1], Krisztina Ludányi[1,*]

1 Department of Pharmaceutics, Semmelweis University, Hőgyes Endre utca 7., H-1092, Budapest, Hungary; virag.david@pharma.semmelweis-univ.hu (D.V.); famkremmer@gmail.com (T.K.); gyorizsofireka@gmail.com (Z.H.); kiss.borbala@pharma.semmelweis-univ.hu (B.D.K.); antal.istvan@pharma.semmelweis-univ.hu (I.A.)

2 Department of Dermatology, Venereology and Dermatooncology, Semmelweis University, Mária utca. 41., H-1085, Budapest, Hungary; lorincz.kende@med.semmelweis-univ.hu (K.L.); kiss.norbert@med.semmelweis-univ.hu (N.K.); jobbagyantal@gmail.com (A.J.); bozsanyi.szabolcs@med.semmelweis-univ.hu (S.B.); gulyaslili1998@gmail.com (L.G.); wikonkal.norbert@med.semmelweis-univ.hu (N.W.)

3 MTA-ELTE Lendület Ion Mobility Mass Spectrometry Research Group, ELTE Eötvös Loránd University, Faculty of Science, Institute of Chemistry, Pázmány Péter sétány 1/A, H-1117, Budapest, Hungary; gitta.schlosser@ttk.elte.hu (G.S.); adina.borbely@ttk.elte.hu (A.B.)

* Correspondence: ludanyi.krisztina@pharma.semmelweis-univ.hu

Supplementary Materials

Table S1. The glycan isomers identified in the melanoma and control samples. Retention time, mass accuracy, sialic acid linkage type and fucose positions (where available) are also given. In the glycan compositions N refers to N-acetylglucosamine, H to hexoses (galactose and mannose), S to sialic acid and F to fucose units. The digits after the letters refer to the number of each residue build up the molecule.

Glycan	Isomer	RT (min)	Mass error (ppm)	Sialic acid linkage	Fucose position
**Biantennary**					
**N4H5SF**	Isomer 1	43.49	2.57	α-2,3	core
	Isomer 2	44.71	-3.04	-	-
	Isomer 3	45.33	3.90	α-2,6	core
	Isomer 4	46.43	4.32	-	-
	Isomer 5	47.11	3.56	α-2,6	antenna
**N4H5S2**	Isomer 1	70.53	2.86	α-2,3	-
	Isomer 2	73.02	3.00	α-2,3, α-2,6	-
	Isomer 3	75.31	2.92	α-2,6	-
**N4H5S2F**	Isomer 1	67.93	7.40	α-2,3	core
	Isomer 2	69.26	13.22	-	-
	Isomer 3	69.99	8.32	α-2,3, α-2,6	core
	Isomer 4	70.60	12.73	-	-
	Isomer 5	71.16	9.39	-	-
	Isomer 6	72.28	8.57	α-2,3, α-2,6	antenna
	Isomer 7	73.50	14.75	-	-
**Triantennary**					
**N5H6S**	Isomer 1	43.94	4.07	-	-
	Isomer 2	44.90	4.16	-	-
	Isomer 3	45.27	3.86	α-2,6	-
	Isomer 4	46.06	5.95	-	-
	Isomer 5	46.67	4.17	α-2,6	-
	Isomer 6	47.11	1.79	α-2,6	-
**N5H6S2**	Isomer 1	65.94	1.56	-	-
	Isomer 2	66.80	2.57	-	-
	Isomer 3	67.41	2.15	-	-
	Isomer 4	68.57	0.48	α-2,6	-
	Isomer 5	69.39	1.41	α-2,6	-
	Isomer 6	70.06	2.46	α-2,6	-
	Isomer 7	71.13	2.51	-	-
	Isomer 8	71.72	1.94	-	-
**N5H6S2F**	Isomer 1	63.78	-4.98	-	-
	Isomer 2	66.15	-5.16	-	-
	Isomer 3	65.98	-4.87	-	-
	Isomer 4	67.62	0.02	α-2,6	antenna
	Isomer 5	69.32	0.48	α-2,6	antenna
	Isomer 6	70.92	1.01	-	-
**N5H6S3**	Isomer 1	106.35	2.74	-	-
	Isomer 2	108.49	4.05	α-2,6	-
	Isomer 3	111.10	1.73	α-2,6	-
**N5H6S3F**	Isomer 1	101.56	0.16	α-2,3, α-2,6	antenna
	Isomer 2	104.04	0.76	α-2,3, α-2,6	antenna
	Isomer 3	106.62	-3.14	-	-

**N5H6S3F2**	Isomer 1	96.54	6.62	-	-
	Isomer 2	99.08	3.39	-	-
	Isomer 3	102.40	3.61	-	-
	Isomer 4	106.30	-3.49	-	-
	Isomer 5	108.70	-4.46	-	-
	Isomer 6	112.60	0.43	-	-
**N5H6S3F3**	Isomer 1	94.10	-9.58	-	-
	Isomer 2	102.00	-8.98	-	-
	Isomer 3	104.23	-7.08	-	-
	Isomer 4	108.77	-6.81	-	-
**Tetraantennary**					
**N6H7S**	Isomer 1	44.44	0.97	-	-
	Isomer 2	45.06	-0.35	-	-
	Isomer 3	45.43	1.75	-	-
	Isomer 4	45.86	1.71	-	-
	Isomer 5	46.52	1.73	-	-
**N6H7SF**	Isomer 1	43.62	2.11	-	-
	Isomer 2	44.17	-1.30	-	-
	Isomer 3	44.56	8.65	-	-
	Isomer 4	44.94	4.29	-	-
	Isomer 5	45.66	2.93	-	-
	Isomer 6	46.38	2.44	-	-
	Isomer 7	47.04	6.94	-	-
**N6H7S2**	Isomer 1	63.28	4.27	-	-
	Isomer 2	65.07	4.33	-	-
	Isomer 3	65.66	3.32	α-2,3, α-2,6	-
	Isomer 4	66.83	3.94	α-2,3, α-2,6	-
	Isomer 5	67.54	3.97	α-2,6	-
**N6H7S2F**	Isomer 1	64.33	3.39	α-2,6	antenna
	Isomer 2	65.01	3.23	α-2,6	antenna
	Isomer 3	66.00	2.83	α-2,6	antenna
	Isomer 4	66.76	3.30	α-2,6	antenna
	Isomer 5	67.32	5.37	-	-
**N6H7S3**	Isomer 1	92.20	-0.91	-	-
	Isomer 2	94.31	1.65	-	-
	Isomer 3	95.87	1.07	-	-
	Isomer 4	96.76	-3.32	α-2,3, α-2,6	-
	Isomer 5	97.99	-3.50	α-2,3, α-2,6	-
	Isomer 6	99.27	-3.88	α-2,3, α-2,6	-
	Isomer 7	101.17	-2.98	α-2,6	-
**N6H7S3F**	Isomer 1	87.74	5.42	-	-
	Isomer 2	90.29	5.98	-	-
	Isomer 3	93.38	6.09	α-2,6	antenna
	Isomer 4	94.88	3.66	α-2,6	antenna
	Isomer 5	96.18	1.19	α-2,3, α-2,6	antenna
	Isomer 6	97.68	7.11	α-2,6	antenna
	Isomer 7	99.85	6.22	-	-
**N6H7S4**	Isomer 1	87.44	2.87	α-2,6	-
	Isomer 2	89.74	-3.40	-	-

	Isomer 3	103.90	-2.97	-	-
**N6H7S4F**	Isomer 1	83.46	1.81	-	-
	Isomer 2	93.77	3.25	-	-
**N6H7S4F2**	Isomer 1	99.39	3.67	-	-
	Isomer 2	108.76	5.36	-	-
**N6H7S4F3**	Isomer 1	90.15	-3.56	-	-
**N7H8S3**	Isomer 1	85.61	-1.5	-	-
	Isomer 2	88.24	4.83	-	-
	Isomer 3	90.28	1.73	-	-
	Isomer 4	91.09	-1.17	-	-
	Isomer 5	92.49	-1.44	α-2,6	-
	Isomer 6	93.73	-1.49	α-2,6	-
	Isomer 7	95.27	-5.03	-	-

Table S2. Clinical characteristics of the melanoma patients. Staging was based on the 8th edition of the American Joint Committee on Cancer (AJCC) tumor, node, metastasis (TNM) classification system. Breslow thickness refers to the depth of the melanoma from the surface of the skin to the deepest point of the tumor measured under microscope. pT3: Breslow thickness of the primary tumor is between 2.0 and 4.0 mm, which is subdivided into T3a or T3b based upon the absence or presence of ulceration. pT4: Breslow thickness of the primary tumor is more than 4.0 mm, which is subdivided into T4a or T4b based upon the absence or presence of ulceration. NMM refers to nodular malignant melanoma and SSM to superficial spreading melanoma.

Cases	Status	Breslow thickness (mm)	S100B serum level (µg/L)
1	MM (non classified) (pT4b)	4.53	0.05
2	NMM (pT3b)	3.28	0.06
3	NMM (pT4a)	7.70	0.06
4	SSM with secunder nodular component (pT4b)	5.50	0.04
5	NMM (pT4b)	9.30	0.16
6	MM (non classified) (pT3b)	2.54	0.05
7	SSM (pT4a)	4.80	0.06
8	SSM (pT4a)	6.30	0.02
9	NMM (pT4b)	5.15	0.13
10	MM (non classified) (pT3b)	2.40	0.34
11	NMM (pT4b)	11.00	0.80
12	NMM (pT4b)	13.00	1.90
13	SSM (pT3b)	3.93	0.04
14	NMM (pT4b)	7.50	0.05
15	NMM (pT4a)	4.50	22.24
16	NMM (pT4a)	5.50	1.33
17	NMM (pT4b)	7.60	0.08
18	NMM (pT4b)	4.33	0.03

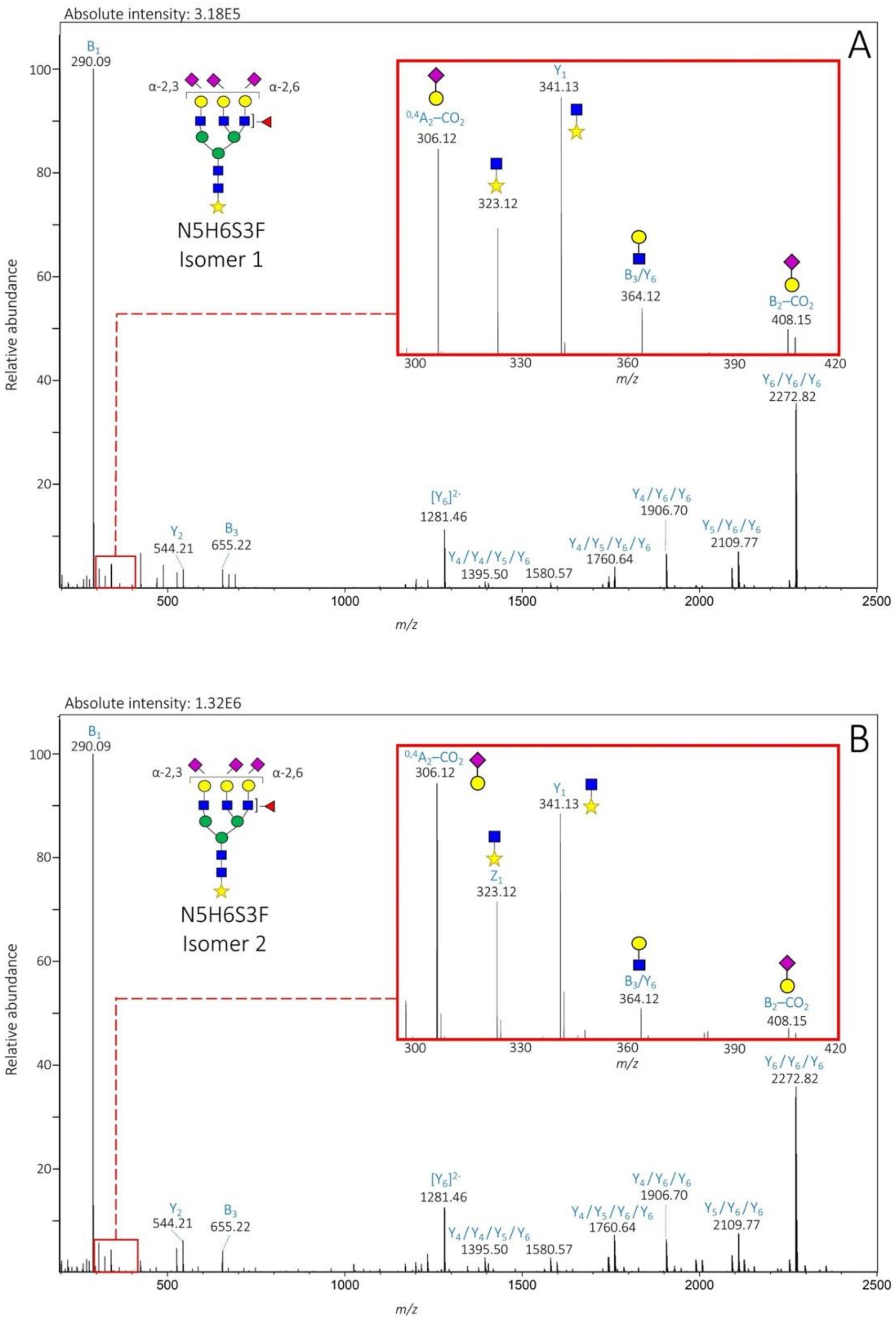

Figure S1. MS/MS spectra of isomers 1 (A) and 2 (B) of triantennary glycan N5H6S3F.
Both spectra contain diagnostic fragment ions *m/z* 306.12 and *m/z* 408.15 indicating the presence of α-2,6 and α-2,3 sialic acids. An increase in the ratio of α-2,6 to α-2,3 can be obeserved on the second spectra suggesting that two out of three sialic acids are α-2,3 linked in isomer 1 and two out of three are α-2,6 linked in isomer 2.

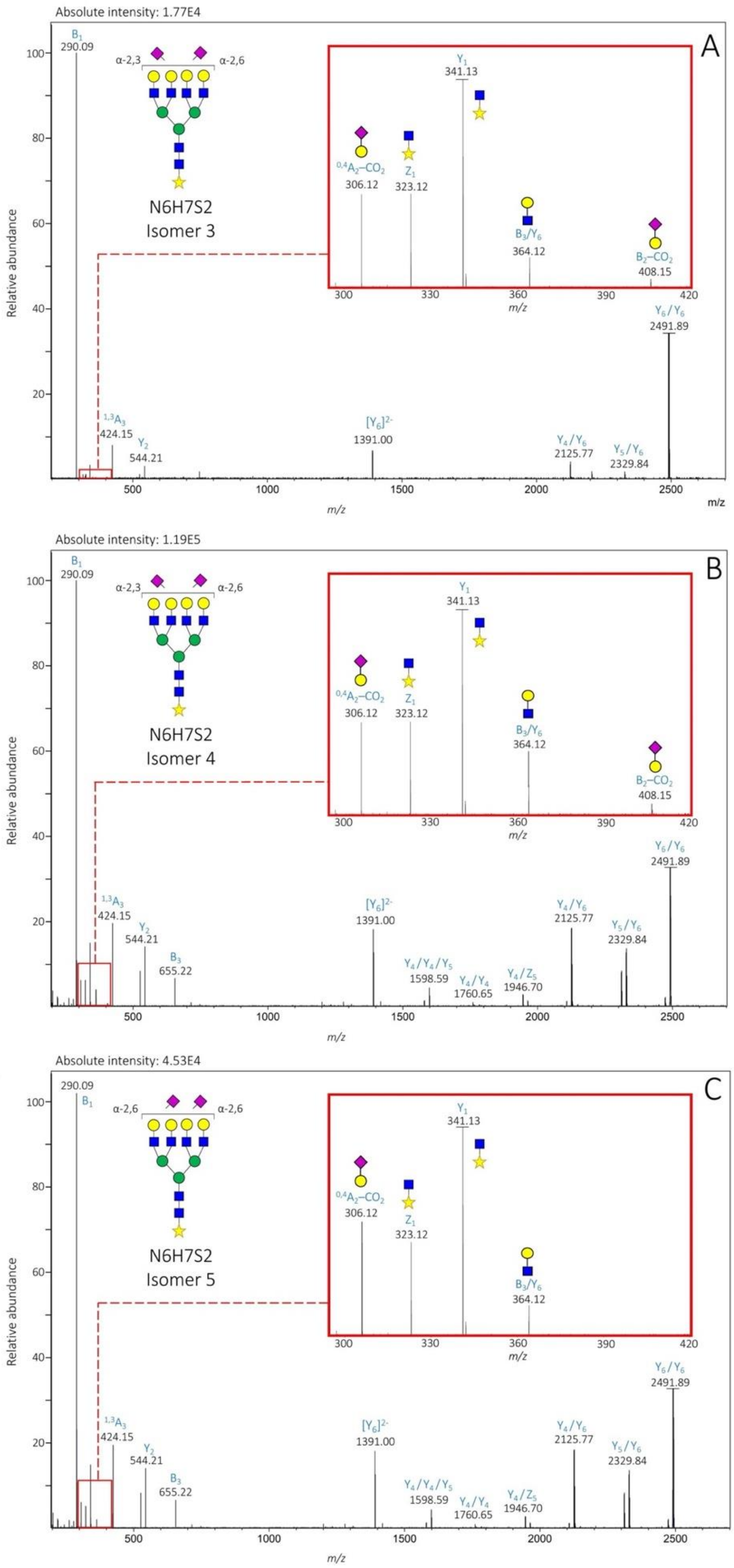

Figure S2. MS/MS spectra of isomers 3 (A), 4 (B) and 5 (C) of tetraantennary glycan N6H7S2.

Isomers 3 and 4 contain diagnostic fragment ions *m/z* 306.12 and *m/z* 408.15 indicating the presence of α-2,6 and α-2,3 linked sialic acids. The spectrum of isomer 5 lacks *m/z* 408.15 demonstrating that it is α-2,6 sialylated only.
